# A case report of carcinoma of the papilla of Vater associated with a hyperplasia–dysplasia–carcinoma sequence by pancreaticobiliary maljunction

**DOI:** 10.1186/s12957-024-03347-z

**Published:** 2024-02-22

**Authors:** Takahiro Korai, Yasutoshi Kimura, Kazunori Watanabe, Siew-Kee Low, Masafumi Imamura, Minoru Nagayama, Kazuharu Kukita, Takeshi Murakami, Toru Kato, Yuta Kondo, Daisuke Kyuno, Taro Sugawara, Ayako Murota, Yujiro Kawakami, Yoshiharu Masaki, Hiroshi Nakase, Ichiro Takemasa

**Affiliations:** 1 Department of Surgery, Surgical Oncology and Science, Sapporo Medical University School of Medicine, 291 Minami-1-jo Nishi 16-chome, Chuo-ku, Sapporo, Hokkaido 060-8543 Japan; 2 Hokkaido Gastroenterological Hospital, Sapporo, Hokkaido Japan; 3 Department of Gastroenterological Surgery II, Faculty of Medicine, Hokkaido University, Sapporo, Hokkaido Japan; 4 Cancer Precision Medicine Center, Japanese Foundation for Cancer Research, Tokyo, Japan; 5 Department of Surgical Pathology, Sapporo Medical University School of Medicine, 291 Minami-1-jo Nishi 16-chome, Chuo-ku, Sapporo, 060-8543 Hokkaido Japan; 6 Department of Gastroenterology and Hepatology, Sapporo Medical University School of Medicine, Sapporo, Hokkaido Japan

**Keywords:** Carcinoma of the papilla of Vater, Pancreaticobiliary maljunction, Hyperplasia, Dysplasia, Next-generation sequencing, Liquid biopsy, Formalin-fixed paraffin-embedded, *ERBB2*, cfDNA, ctDNA

## Abstract

**Background:**

Pancreaticobiliary maljunction (PBM) is a known risk factor for biliary tract cancer. However, its association with carcinoma of the papilla of Vater (PVca) remains unknown. We report a case with PVca that was thought to be caused by the hyperplasia–dysplasia–carcinoma sequence, which is considered a mechanism underlying PBM-induced biliary tract cancer.

**Case presentation:**

A 70-year-old woman presented with white stool and had a history of cholecystectomy for the diagnosis of a non-dilated biliary tract with PBM. Esophagogastroduodenoscopy revealed a tumor in the papilla of Vater, and PVca was histologically proven by biopsy. We finally diagnosed her with PVca concurrent with non-biliary dilated PBM (cT1aN0M0, cStage IA, according to the Union for International Cancer Control, 8th edition), and subsequently performed subtotal stomach-preserving pancreaticoduodenectomy. Pathological findings of the resected specimen revealed no adenomas and dysplastic and hyperplastic mucosae in the common channel slightly upstream of the main tumor, suggesting a PBM related carcinogenic pathway with hyperplasia–dysplasia–carcinoma sequence. Immunostaining revealed positivity for CEA. CK7 positivity, CK20 negativity, and MUC2 negativity indicated that this PVca was of the pancreatobiliary type. Genetic mutations were exclusively detected in tumors and not in normal tissues, and bile ducts from formalin-fixed paraffin-embedded samples included mutated-*ERBB2* (Mutant allele frequency, 81.95%). Moreover, of the cell-free deoxyribonucleic acid (cfDNA) extracted from liquid biopsy mutated-*ERBB2* was considered the circulating-tumor deoxyribonucleic acid (ctDNA) of this tumor.

**Conclusions:**

Herein, we report the first case of PVca with PBM potentially caused by a “hyperplasia–dysplasia–carcinoma sequence” detected using immunostaining and next-generation sequencing. Careful follow-up is required if pancreaticobiliary reflux persists, considering the possible development of PVca.

**Supplementary Information:**

The online version contains supplementary material available at 10.1186/s12957-024-03347-z.

## Background

Pancreaticobiliary maljunction (PBM) is a congenital anomaly defined as the union of the pancreatic and biliary ducts outside the duodenal wall, thus causing pancreaticobiliary reflux [1]. The incidence of biliary tract cancer in patients with a non-dilated biliary tract with concomitant PBM was 42.4%, and cancer localization was 88% for gallbladder cancer and 7% for cholangiocarcinoma [2]. Therefore, cholecystectomy is recommended in many cases of non-dilated biliary tract with PBM; however, there is no consensus regarding extrahepatic bile duct resection. Residual bile duct cancer has been reported to be detectable during long-term follow-up, even after termination of pancreaticobiliary reflux [3, 4], and 23 (1.8%) of 1291 patients developed residual bile duct cancer after cyst excision [4]. In addition to residual bile duct cancer, carcinoma of the papilla of Vater (PVca) has been reported; however, it is considerably rare [5, 6]. Although the risk factors for PVca have not been clarified [7], we report our experience with PVca that was thought to be caused by the hyperplasia–dysplasia–carcinoma sequence, which is considered a mechanism underlying PBM-related biliary tract cancer. This is the first report of such a case, which was confirmed not only by immunohistochemical examination but also by genetic analysis using next-generation sequencing (NGS) of formalin-fixed paraffin-embedded (FFPE) samples and liquid biopsy (LB) specimens.

## Case presentation

### Case

**Fig. 1:**
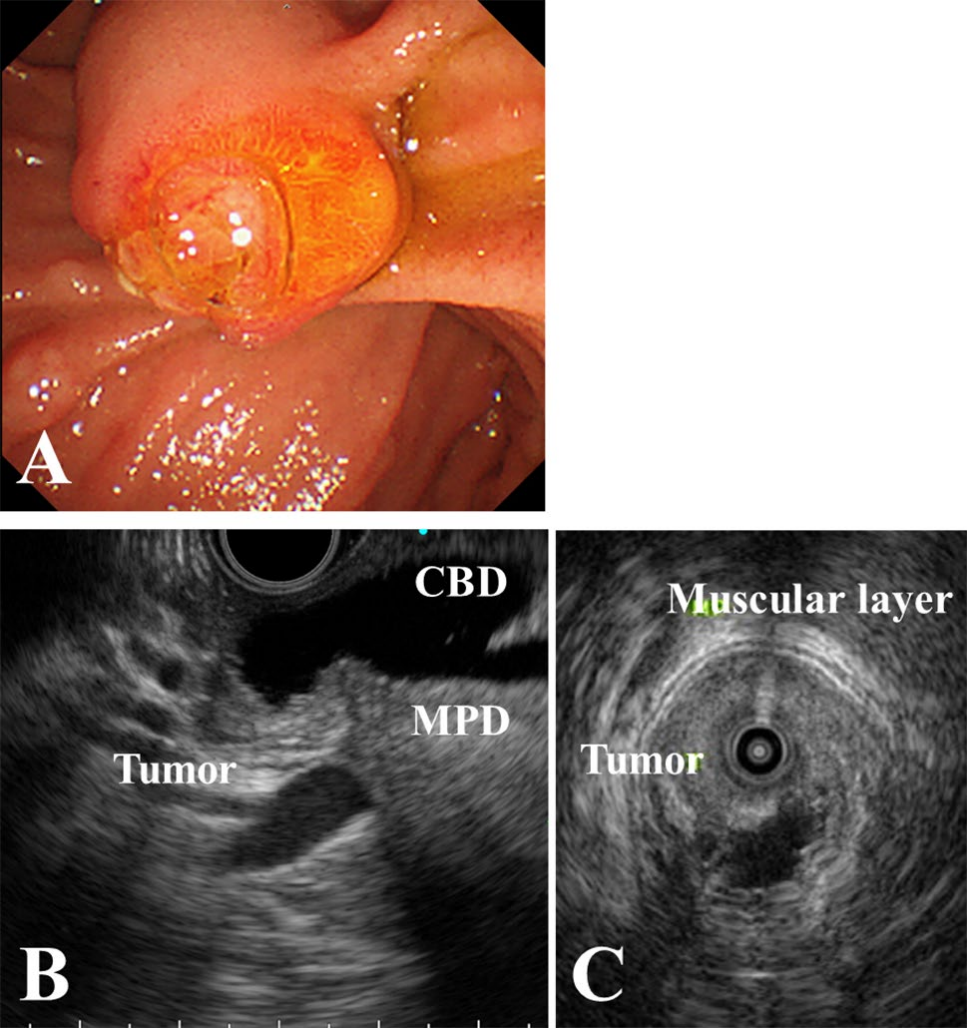
Endoscopy and echography. (**A**) Esophagogastroduodenoscopy revealed a tumor of the papilla of Vater. (**B**, **C**) Endoscopic ultrasound and intraductal ultrasonography revealed that the tumor was located in the common channel and demonstrated no invasion of the duodenal muscular layer and pancreas. CBD, Common bile duct; MPD, Main pancreatic duct

**Fig. 2:**
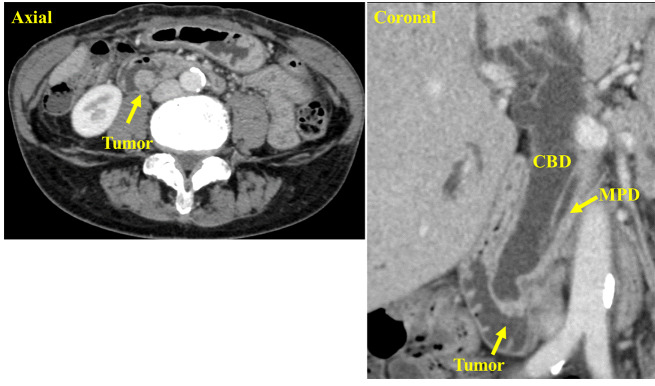
Abdominal enhanced computed tomography (CT). CT revealed a tumor in the duodenum, with a size of 14 × 14 mm (arrow). CBD, Common bile duct; MPD, Main pancreatic duct

**Fig. 3:**
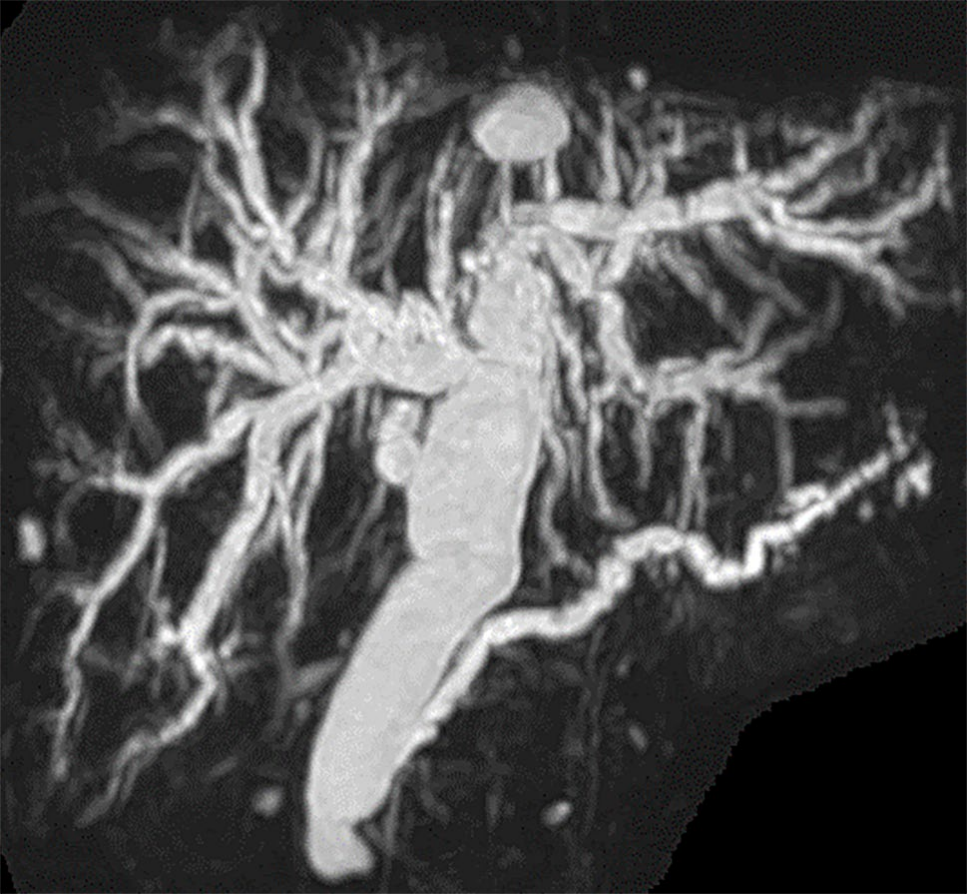
Magnetic resonance choledochopancreatography (MRCP). MRCP revealed dilatation of the extra/intrahepatic bile duct and main pancreatic duct, and the length of the common channel was 23 mm

A 70-year-old woman presented with white stool and the patient was referred to our hospital for further investigation of jaundice. The patient had undergone cholecystectomy for the diagnosis of a non-dilated biliary tract with PBM (P-C type) approximately 30 years prior at another hospital. On physical examination, the patient’s abdomen was soft, and no mass was palpated. Laboratory data on admission revealed high levels of carcinoembryonic antigen (6.1 ng/mL), while carbohydrate antigen 19 − 9 was within normal ranges. Esophagogastroduodenoscopy revealed a tumor in the papilla of Vater, and the histological examination of the biopsy specimens revealed adenocarcinoma. (Fig. 1A). Endoscopic ultrasound and intraductal ultrasonography showed that the tumor was located in the common channel with no invasion to the sphincter of Oddi, duodenal muscular layer, or pancreas (Fig. 1B and C). Abdominal enhanced computed tomography (CT) revealed a 14 × 14 mm tumor in the duodenum. No enlarged lymph nodes or distant metastases were observed (Fig. 2). Magnetic resonance cholangiopancreatography demonstrated dilatation of the extra/intrahepatic bile duct and main pancreatic duct; the length of the common channel was 23 mm (Fig. 3). We finally diagnosed her with PVca with a non-biliary dilated PBM (cT1aN0M0, cStage IA, according to the Union for International Cancer Control [UICC], 8th edition), and subtotal stomach-preserving pancreaticoduodenectomy was performed.

### Surgical procedures

A median incision was placed in the upper abdomen. The pancreas was dissected at the anterior surface of the superior mesenteric vein. The modified Child’s reconstruction procedures were performed, and the remnant pancreas was anastomosed with the jejunal limb using the modified Blumgart method. Neither peritoneal dissemination nor lymph node metastases were detected during surgery. The regional lymph nodes of the papillary carcinoma were dissected. The operation lasted 370 min, and the estimated blood loss was 15 mL. No intraoperative blood transfusions were required.

### Postoperative course

The postoperative course was uneventful, and the patient was discharged on the 24th postoperative day. The patient was recurrence-free for 4 years after surgery.

### Macroscopic and pathological findings of the resected specimen

**Fig. 4:**
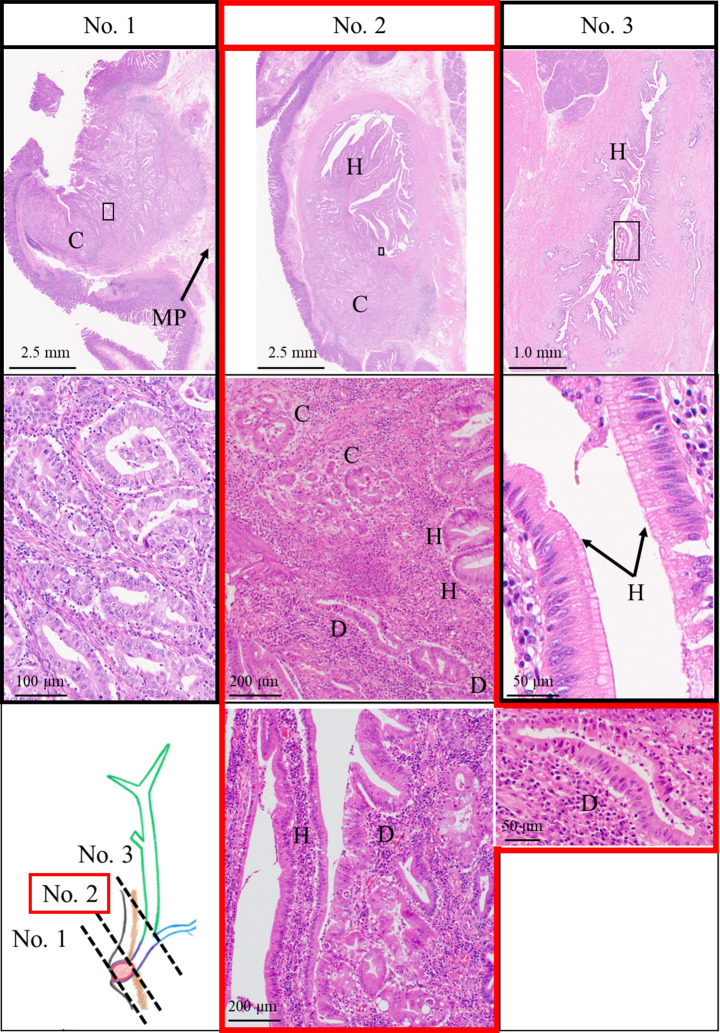
Morphological evaluation derived from pathological findings. No adenomas were observed, suggesting a hyperplasia–dysplasia–carcinoma sequence carcinogenic mechanism. No. 1: Main part of the carcinoma (C) in the common channel. No. 2: The depiction of front between hyperplasia (H) area, dysplasia (D) area, and carcinoma (C) area is observed in the common channel. No. 3: Hyperplasia (H) in the common bile duct. There was no evidence of increased nuclear-to-cytoplasmic ratio, increased nuclear chromatin and loss of nuclear polarity, or cell overlap. MP, Muscularis propria; Panc., Pancreas

**Fig. 5:**
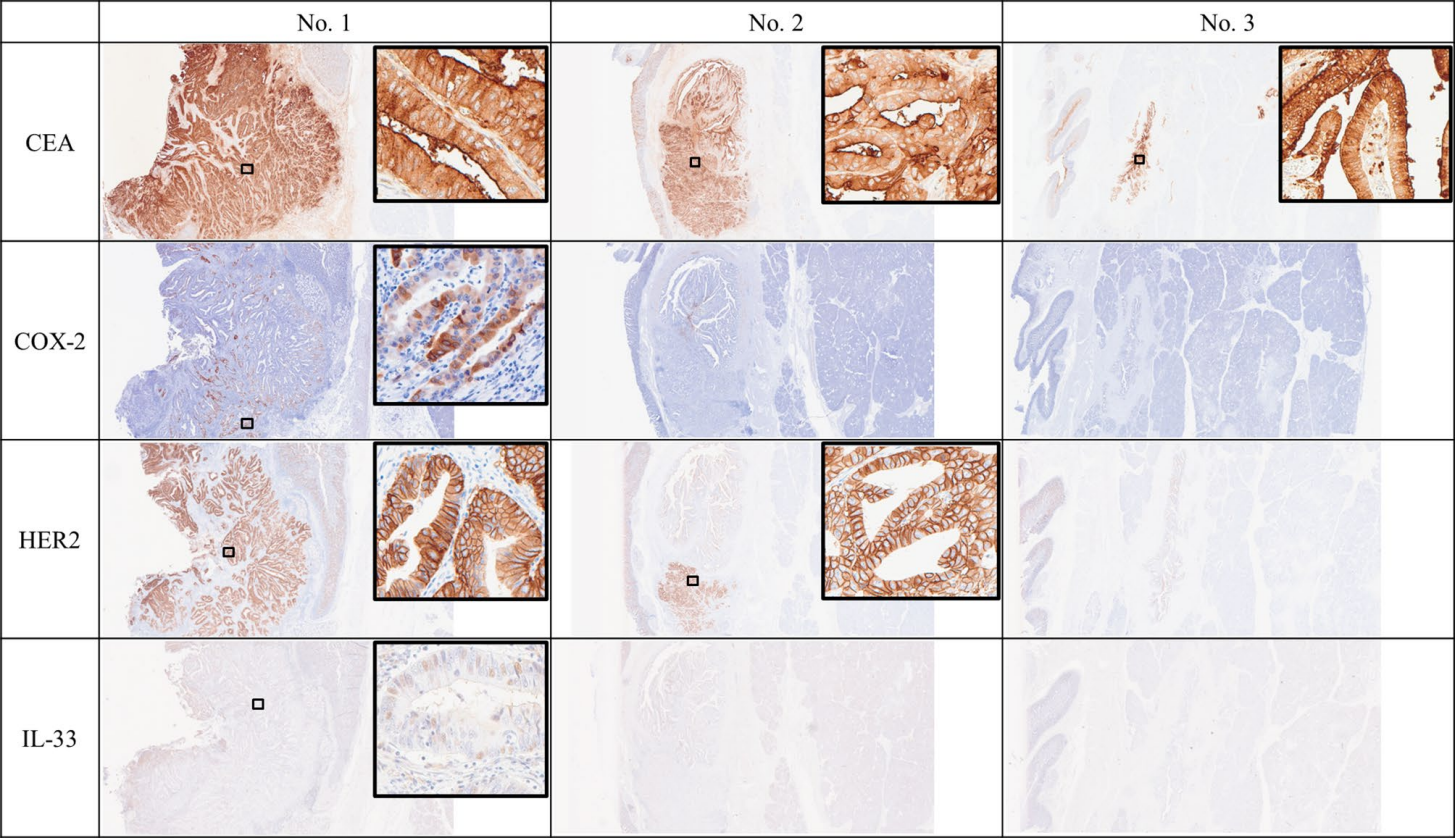
Immunohistological findings of the resected specimen. CEA was highly expressed at all sites of the hyperplasia, dysplasia, and carcinoma. COX-2, HER2, and IL-33 expression were positive in the carcinoma tissues

This was a PBM case without biliary dilation (P-C type), the tumor was diagnosed as PVca developing from the epithelium of the common channel, and the tumor diameter was 9 × 8 mm. The tumor invaded the Oddi sphincter and submucosa but did not invade the muscularis propria of the duodenum (No. 1, Fig. 4). The pathological diagnosis was pT1bN1M0 pStage IIIA, according to the UICC, because of the presence of a positive lymph node (2/37 lymph nodes). A front was observed between hyperplasia and dysplasia areas within the mucosal epithelium of the common channel, slightly upstream of the main tumor (No. 2, Fig. 4). At the hyperplastic area, there was no evidence of increased nuclear-to-cytoplasmic ratio, increased nuclear chromatin and loss of nuclear polarity, or cell overlap (No. 2 and No.3, Fig. 4, Supplement Fig. 1). In addition, at the dysplastic area, the findings of disturbed polarity, increased nuclear chromatin, and increased nuclear-to-cytoplasmic ratio suggested that the tumor was equivalent to BilIN-3 (high grade dysplasia) (No. 2, Fig. 4). A hyperplastic mucosa was found throughout the common bile duct (No. 3, Fig. 4). Immunostaining revealed positivity for CEA, COX-2, HER2, and IL-33 in the carcinoma (Fig. 5). CK7 and MUC1 positivity; MUC5 partial positivity; CDX2 and MUC2 negativity; and mostly CD20 negativity indicated that this PVca was of the pancreatobiliary type, not gastric type. In addition to MUC6 negativity in the carcinoma area, CDX2 was also negative, thereby we did not determine the lesion to be of the intestinal type [8]. p53 was wild-type immunostaining pattern (Supplement Fig. 2).

### Genetic analysis using FFPE and LB

#### 1–1 Cell-free total nucleic acid (cfTNA) and genomic DNA extraction

Thirteen plasma samples were collected between July 2019 and September 2020. Cell-free total nucleic acid (cfTNA) was extracted using the MagMAX™ Cell-Free Total Nucleic Acid Isolation Kit (Thermo Fisher Scientific) or the NextPrep-Mag™ cfDNA Automated Isolation Kit (PerkinElmer), according to the manufacturers’ protocols. Genomic DNA was extracted from the buffy coat using the FlexiGene DNA Kit (Qiagen).

Ten 5-µm slices of FFPE slides were used to extract genomic deoxyribonucleic acid (DNA). Genomic DNA from the tumor tissue, common bile duct, and normal tissue was extracted using a GeneRead™ DNA FFPE Kit (Qiagen), according to the manufacturer’s protocol. The extracted cfTNA and genomic DNA were quantified using the Qubit™ DNA (High Sensitivity) Assay Kit and Qubit DNA (Broad Range) Assay Kit (Thermo Fisher Scientific), respectively. The quality and size of the extracted cfTNA were evaluated with the High-Sensitivity D5000 ScreenTape Assay (Agilent) and the quality of genomic DNA was evaluated with the Genomic DNA ScreenTape Assay (Agilent), using TapeStation (Agilent).

#### 1–2 Library construction

The NGS library was constructed using the Oncomine™ Pan-Cancer Cell-Free Assay (Thermo Fisher Scientific), according to the manufacturer’s protocol. Libraries were constructed using 12.3–20 ng of cfTNA, and 30 ng of genomic DNA from buffy coat.

Regarding tumor tissue, libraries were prepared using 40 ng of extracted genomic DNA from FFPE using the Ion AmpliSeq™ Comprehensive Cancer Panel (Thermo Fisher Scientific) with Ion Xpress™ Barcode Adapters (Thermo Fisher Scientific), according to the manufacturer’s protocol (Thermo Fisher Scientific). The quality of all constructed libraries was evaluated with a High-Sensitivity D1000 ScreenTape (Agilent), using TapeStation (Agilent).

#### 1–3 Targeted NGS

The constructed libraries were subjected to template preparation using the Ion Chef™ System (Thermo Fisher Scientific) with either the Ion 540 Chef Kit (Thermo Fisher Scientific) or the Ion 550 Chef Kit (Thermo Fisher Scientific). Thereafter, sequencing was performed using the Ion GeneStudio™ S5 Prime System (Thermo Fisher Scientific).

#### 1–4 Sequencing data analysis

Sequence alignment with hg19 as the reference genome and variant calling were performed using the Torrent Suite Software v5.16 and Ion Reporter v5.16 and v5.18. The workflows used for analyses included Oncomine TagSeq Pan-Cancer Liquid Biopsy w2.5 for cfDNA as well as buffy coat, and AmpliSeq CCP w1.2 Tumor–Normal pair for tumor genomic DNA with default parameters. The cutoff for variant calling in cfDNA was 0.065%. Regarding tumor-tissue alterations, mutations with a mutant allele frequency ≥ 5% were considered positive after excluding variants (single-nucleotide polymorphisms) detected in normal tissue. Mutations detected in the buffy coat that were also detected in the plasma cfTNA were evaluated as clonal hematopoiesis-associated mutations [9, 10].

#### 1–5 Results of genetic analysis

Genetic mutations with single nucleotide variants exclusively detected in tumors and not in normal tissue and bile ducts from FFPE specimens included *ERBB2* (Mutant allele frequency; MAF, 81.95%), *POU5F1* (MAF, 12.43%), *FLT1* (MAF, 9.91%), *NCOA2* (MAF, 8.00%), and *KMT2D* (MAF, 7.14%). *ERBB2* was also detected as a genetic mutation with copy number variant present exclusively in tumors compared to normal tissue, with a copy number of nine (Supplement table). The genetic mutations with single nucleotide variants detected in bile ducts included *KIT* (MAF, 11.03%) (Table 1). NGS identified no genetic abnormalities in *p53*. Considering the immunostaining results, this case was considered wild-type for *p53*. The cell-free DNA (cfDNA) (baseline) obtained from preoperative plasma was *ERBB2* (MAF, 0.24%) and *ERBB2* was never detected after surgery (Supplement Fig. 3).

**Table 1:** Genetic mutations with single nucleotide variant of mutant allele frequency > 5% detected only in tumors and bile ducts compared to normal tissue

					MAF		
Genes	Amino acid	Type	Reference	Allele	Tumor (Carcinoma)	Bile duct (Hyperplasia)	Normal tissue (Pancreas)
*ERBB2*	p.S310Y	SNV	C	A, T	81.95%		
*POU5F1*	p.R201Q	SNV	C	T	12.43%		
*FLT1*	p.D974N	SNV	C	T	9.91%		
*NCOA2*	p.E189K	SNV	C	T	8.00%		
*KMT2D*	p.P3621R	SNV	G	C	7.14%		
*EZH2*	p.R313Q	SNV	C	T	6.73%		
*CSMD3*	p.R2944T	SNV	C	G	6.60%		
*TSC2*	p.S1132L	SNV	C	T	6.20%		
*MARK1*	p.R771H	SNV	G	A	6.02%		
*AKAP9*	p.S3767L	SNV	C	T	5.51%		
*NUP214*	p.V1720A	SNV	T	C	5.26%		
*RB1*	p.V735I	SNV	G	A	5.08%		
*KIT*	p.H263Q	SNV	T	G		11.03%	
*ERBB4*	p.R488Q	SNV	C	T		7.75%	
*MITF*	p.S258L	SNV	C	T		7.61%	
*UBR5*	p.I810V	SNV	T	C		5.77%	
*USP9X*	p.R2551Q	SNV	G	A		5.19%	

SNV, single nucleotide variant; MAF, Mutant allele frequency

## Discussion and conclusions

We reported the first case of PVca after cholecystectomy for PBM with a non-dilated biliary tract potentially caused by a hyperplasia–dysplasia–carcinoma sequence detected using detailed immunostaining and NGS. Careful follow-up is needed after cholecystectomy for patients with PBM with a non-dilated biliary tract due to the possibility of carcinogenesis from the duodenal papillary region and conventional biliary carcinogenesis.

The incidence of biliary tract cancer in patients with PBM with a non-dilated biliary tract has been reported as 42.4%, and 88% of biliary tract cancer is localized in the gallbladder cancer while 7% is classified as cholangiocarcinoma [2]. Therefore, cholecystectomy is often recommended for patients with PBM with a non-dilated biliary tract; however, there is no consensus regarding extrahepatic bile duct resection [11, 12]. The estimated incidence of cancer development after a diversion operation for congenital biliary dilatation is 0.7–5.4%, and the interval between the operation and cancer detection ranges from one to 19 years [3, 4]. However, as there are no reports regarding the incidence of biliary tract cancer in residual bile ducts after bile duct resection or after cholecystectomy in patients with PBM with a non-dilated biliary tract, it is unclear whether bile duct resection is a good treatment option. It is presumed that the reflux of pancreatic juice into the bile duct persists as the common bile duct and papilla are preserved after cholecystectomy. Therefore, the mucosal damage in the common duct is considered to be persistent. Previous studies have focused on PVca with PBM, though it remains unclear whether PBM is the cause of carcinogenesis in any of the previous studies [5, 6, 13–17]. PVca is classified as cholangiocarcinoma, and the usual carcinogenic process underlying PVca is the adenoma–carcinoma sequence [18]. While the hyperplasia–dysplasia–carcinoma sequence has been proposed as a carcinogenic process in the context of PBM [19, 20], there are no reports of an association between PBM and PVca. Based on the morphological, pathological, and genetic analyses presented in this study, this is the first report of PVca thought to be caused by the hyperplasia–dysplasia–carcinoma sequence.

No previous reports have discussed the risk factors for PVca or the relationship between PBM and PVca. The pathological findings in this study suggest morphological carcinogenesis by the hyperplasia–dysplasia–carcinoma sequence. Although the usual carcinogenic process underlying PVca is the adenoma–carcinoma sequence [18], no adenoma was observed in this patient. Furthermore, hyperplasia was observed throughout the bile duct and dysplasia was observed in the vicinity of the carcinoma. We could not locate a “clear” front on any of the intercepts available. However, we successfully identified a transition from hyperplasia to dysplasia within mucosal surface epithelium, albeit not within the same glandular duct. If the sections had been longitudinally oriented along the bile duct, it might have facilitated a clearer delineation of the specific boundaries. However, we did not discern any evident transition from hyperplasia to dysplasia or from dysplasia to carcinoma within the same glandular duct. Our experience with these cases has prompted us to reconsider our methodology for specimen preparation in cases of cholangiocarcinoma. Also, it is very interesting that the carcinoma was positive for HER2 expression. The copy number of *ERBB2* in this particular case was found to be nine. The genetic analysis revealed that *ERBB2* was amplified, and thus the resulting abnormal production of the HER2 protein may have contributed to the growth of this tumor. HER2 protein overexpression is caused by *ERBB2* amplification by next-generation sequencing; this has been confirmed in previous reports, and we believe the same to be true in this case [21]. Immunostaining was also positive for CEA in the carcinoma, and COX-2 expression was positive in one region of the carcinoma, as previously reported [22, 23]. Although CD20 partial positivity is not typical, it was determined to be the pancreatobiliary type based on an overall judgment. This was considered to be a finding suggestive of intra-tumor heterogeneity. Since IL-33 overexpression has been reported in gallbladder carcinoma associated with PBM [24], IL-33-positivity of the cancerous area in this patient further suggests that the carcinoma is associated with PBM.

The significance of NGS and liquid biopsy in this case is highlighted by the fact that to date no studies have reported genetic analysis in PVca associated with PBM. Large-scale genome sequencing of PVca arising in an adenoma-carcinoma sequence was conducted in 2016, and these results are already available [25]. Moreover, although there are scattered reports of genetic analysis of gallbladder tissue and cholangiocarcinoma associated with PBM, there are no reports of PVca associated with PBM. In light of the above, the results of the genetic analysis in this case are highly suggestive, and we hope that they will serve as a bridge supporting the accumulation of future cases. *SMAD4* and *TP53* have been reported as genetic abnormalities in cholangiocarcinoma associated with PBM [26, 27]. Large-scale genome sequencing of PVca identified *KRAS* (48%) and *TP53* (56%) as the most frequent mutations, followed by *CTNNB1*, *SMAD4*, *APC*, *ELF3*, *GNAS*, *ERBB2*, *ERBB3*, and *LOXHD1*, with frequencies ranging from 10 to 30% [25]. The pancreatobiliary type, as in this patient, resembles pancreatic cancer, involving *KRAS* (68%), *TP53* (67%), and *SMAD4* (20%) [25]. Genetic analysis of the FFPE samples obtained in this study revealed mutations of *ERBB2*, but no mutations of *KRAS*, *TP53*, or *SMAD4*, which are frequently mutated in patients with pancreatobiliary-type PVca. Of the cfDNA extracted from LB, excluding those detected in the buffy coat and at only one time point during the disease course which may have been in error, *ERBB2* was considered the circulating-tumor DNA (ctDNA) of this tumor. *ERBB2* has been reported as a genetic abnormality in gallbladder cancer with PBM in 17.6% of patients, suggesting that the current patient’s disease may have been associated with PBM [27]. In addition, this tumor demonstrated genetic abnormalities, such as *POU5F1*, *FLT1*, *NCOA2*, and *KMT2D*, which have not been addressed in the large-scale genome sequencing of Vater papillary carcinoma. According to the Catalogue of Somatic Mutations In Cancer (COSMIC) database [28], *KIT* (p.H263Q) unlikely to be a pathogenic mutation. No common pathogenic genetic variants between the bile ducts and the carcinoma were found in this case; however, the presence of a common pathogenic genetic variant would have provided evidence to suggest that the PVca was based on a hyperplasia-dysplasia-carcinoma sequence. A limitation of the present study related to the immunohistological examination and genetic analysis is that the amount of dysplastic tissue was so small that the direct comparison of carcinoma and dysplasia was not possible. In this case, immunohistological examination and genetic analysis were performed as evidence to support the hyperplasia-dysplasia-carcinoma sequence and were compared with previously reported data; however, this comparison was limited to hyperplasia and carcinoma. Considering the amount of dysplastic tissue and the success rate, we did not performed microdissection in this case. Furthermore, the fact that the bile ducts and duodenal papillae were not incised and annular in the resection specimen adversely affected gross morphology and histological evaluation. In other words, it was not possible to present sections with a front between the hyperplasia, dysplasia, and carcinoma areas.

No previous study has reported genetic abnormalities in PVca with PBM, therefore further studies are required to validate these findings.

### Electronic supplementary material

Below is the link to the electronic supplementary material.

Supplementary Material 1: Supplement figure 1: Macroscopic findings of the resected specimen. The sections are aligned in the short-axis direction to the bile duct; carcinoma is found in the papilla of Vater. No. 1?3 used in Figure 4 and 5 correspond to section numbers 33, 31, and 29, respectively.

Supplementary Material 2: Supplement figure 2 Immunohistological all findings of the resected specimen. CK7, MUC1, and MUC5 positivity, CDX2, CK20, and MUC2 negativity indicated that this PVca was of the pancreaticobiliary type. PVca, carcinoma of the papilla of Vater.

Supplementary Material 3: Supplement figure 3 Cell-free DNA obtained from plasma and postoperative changes over time. ERBB2 was never detected after surgery. MAF, Mutant allele frequency

Supplementary Material 4

## Data Availability

All data are provided in the manuscript.

## Abbreviations

PVca: carcinoma of the papilla of Vater
PBM: pancreaticobiliary maljunction
NGS: next-generation sequencing
LB: liquid biopsy
FFPE: formalin-fixed paraffin-embedded
DNA: deoxyribonucleic acid
cfTNA: cell-free total nucleic acid
MAF: Mutant allele frequency
cfDNA: cell-free deoxyribonucleic acid
ctDNA: circulating-tumor deoxyribonucleic acid
