# Supplementary figures and images for: A case report of carcinoma of the papilla of Vater associated with a hyperplasia–dysplasia–carcinoma sequence by pancreaticobiliary maljunction
Source: World J Surg Oncol. 2024 Feb 22;22:63. doi: 10.1186/s12957-024-03347-z (PMC10882841; doi:10.1186/s12957-024-03347-z)

Supplement figure 3


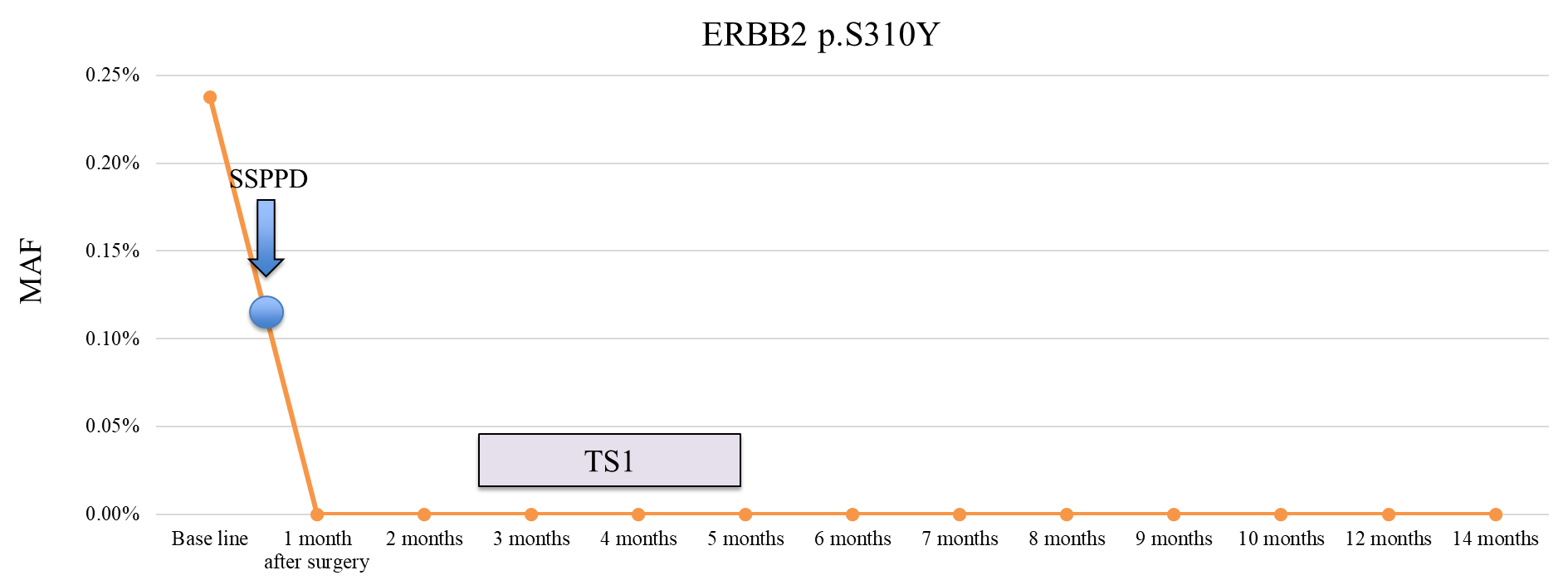

Supplement: Supplementary file 1 — Supplementary Material 1: Supplement figure 1: Macroscopic findings of the resected specimen. The sections are aligned in the short-axis direction to the bile duct; carcinoma is found in the papilla of Vater. No. 1?3 used in Figure 4 and 5 correspond to section numbers 33, 31, and 29, respectively. [file 12957_2024_3347_MOESM1_ESM.docx]

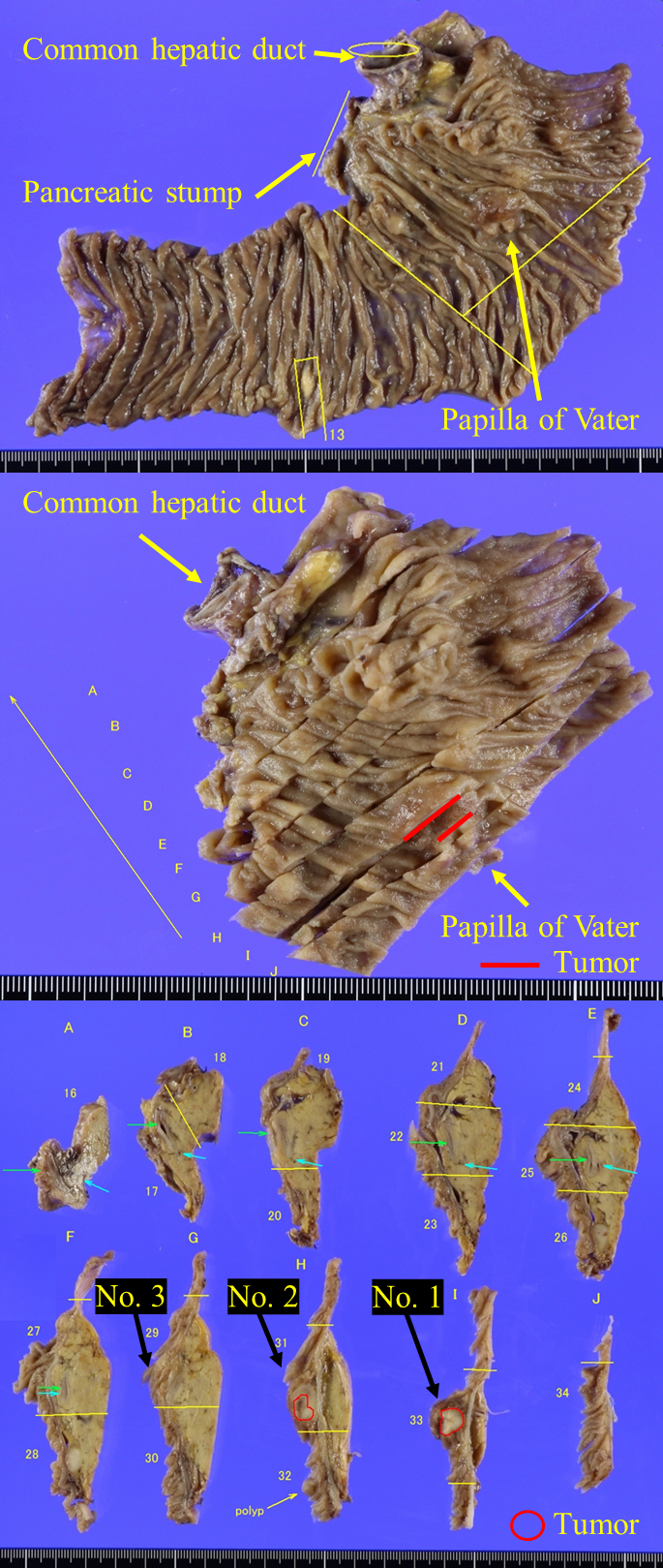
Supplement figure 1

Supplement: Supplementary file 3 — Supplementary Material 3: Supplement figure 3 Cell-free DNA obtained from plasma and postoperative changes over time. ERBB2 was never detected after surgery. MAF, Mutant allele frequency [file 12957_2024_3347_MOESM3_ESM.docx]

Supplement figure 2


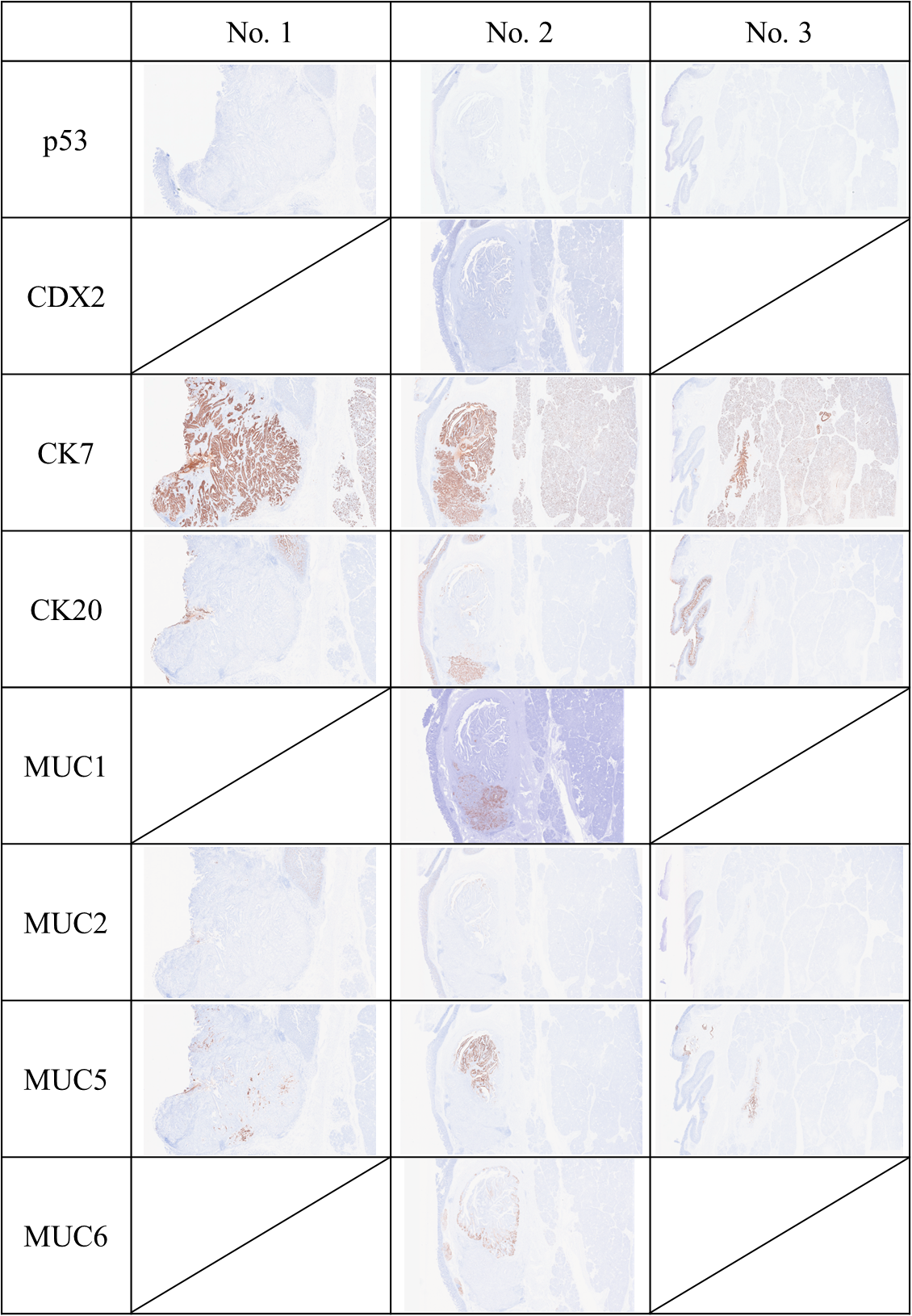

Supplement: Supplementary file 4 — Supplementary Material 4 [file 12957_2024_3347_MOESM4_ESM.docx]
