# Supplementary material for: A case report of carcinoma of the papilla of Vater associated with a hyperplasia–dysplasia–carcinoma sequence by pancreaticobiliary maljunction
Source: World J Surg Oncol. 2024 Feb 22;22:63. doi: 10.1186/s12957-024-03347-z (PMC10882841; doi:10.1186/s12957-024-03347-z)
Supplement: Supplementary file 2 — Supplementary Material 2: Supplement figure 2 Immunohistological all findings of the resected specimen. CK7, MUC1, and MUC5 positivity, CDX2, CK20, and MUC2 negativity indicated that this PVca was of the pancreaticobiliary type. PVca, carcinoma of the papilla of Vater. [file 12957_2024_3347_MOESM2_ESM.docx]

Supplement table

**Genetic mutations with copy number variant only in tumors compared to normal tissue**

|  |  |  |  |  |
| --- | --- | --- | --- | --- |
| Genes | Type | Cytoband | Copy number | Length |
| *ERBB2* | CNV | 17q12 (37687266-37884315) | 9 | 197049 |
| *MIR4728* | CNV | 17q12 (37687266-37884315) | 9 | 197049 |
| *PGAP3* | CNV | 17q12 (37687266-37884315) | 9 | 197049 |
| *CDK12* | CNV | 17q12 (37687266-37884315) | 8 | 197049 |
| *RARA* | CNV | 17q21.2 (38487459-38512488) | 4 | 25029 |
| *RARA-AS1* | CNV | 17q21.2 (38487459-38512488) | 4 | 25029 |
| *DAXX* | CNV | 6p21.32p21.2 (33287152-37141892) | 3 | 3854740 |
| *PIM1* | CNV | 6p21.32p21.2 (33287152-37141892) | 3 | 3854740 |

CNV, copy number variant
